# Factors Associated with Exclusive Breastfeeding of Preterm Infants. Results from a Prospective National Cohort Study

**DOI:** 10.1371/journal.pone.0089077

**Published:** 2014-02-19

**Authors:** Ragnhild Maastrup, Bo Moelholm Hansen, Hanne Kronborg, Susanne Norby Bojesen, Karin Hallum, Annemi Frandsen, Anne Kyhnaeb, Inge Svarer, Inger Hallström

**Affiliations:** 1 Knowledge Centre for Breastfeeding Infants with Special Needs at Department of Neonatology, Copenhagen University Hospital Rigshospitalet, Copenhagen, Denmark; 2 Department of Health Sciences, Faculty of Medicine, Lund University, Lund, Sweden; 3 Danish National Panel of Experts on Breastfeeding Infants with Special Needs; 4 Department of Neonatology, Copenhagen University Hospital Herlev, Herlev, Denmark; 5 Department of Public Health, Section of Nursing, University of Aarhus, Aarhus, Denmark; 6 Department of Neonatology, Viborg Regional Hospital, Viborg, Denmark; 7 Paediatric Department, Holbaek University Hospital, Holbaek, Denmark; 8 Department of Neonatology, Copenhagen University Hospital Hvidovre, Hvidovre, Denmark; 9 Department of Neonatology, Odense University Hospital, Odense, Denmark; The Ohio State Unversity, United States of America

## Abstract

**Background and Aim:**

Evidence-based knowledge of how to guide the mothers of preterm infants in breastfeeding establishment is contradictive or sparse. The aim was to investigate the associations between pre-specified clinical practices for facilitating breastfeeding, and exclusive breastfeeding at discharge as well as adequate duration thereof.

**Methods:**

A prospective survey based on questionnaires was conducted with a Danish national cohort, comprised of 1,221 mothers and their 1,488 preterm infants with a gestational age of 24–36 weeks. Adjusted for covariates, the pre-specified clinical practices were analysed by multiple logistic regression analyses.

**Results:**

At discharge 68% of the preterm infants were exclusively breastfed and 17% partially. Test-weighing the infant, and minimizing the use of a pacifier, showed a protective effect to exclusive breastfeeding at discharge (OR 0.6 (95% CI 0.4–0.8) and 0.4 (95% CI 0.3–0.6), respectively). The use of nipple shields (OR 2.3 (95% CI 1.6–3.2)) and the initiation of breast milk expression later than 48 hours postpartum (OR 4.9 (95% CI 1.9–12.6)) were associated with failure of exclusive breastfeeding at discharge. The clinical practices associated with an inadequate breastfeeding duration were the initiation of breast milk expression at 12–24 hours (OR 1.6 (95% CI 1.0–2.4)) and 24–48 hours (OR 1.8 (95% CI 1.0–3.1)) vs. before six hours postpartum, and the use of nipple shields (OR 1.4 (95% CI 1.1–1.9)).

**Conclusion:**

Early initiation of breast milk pumping before 12 hours postpartum may increase breastfeeding rates, and it seems that the use of nipple shields should be restricted. The use of test-weighing and minimizing the use of a pacifier may promote the establishment of exclusive breastfeeding, but more research is needed regarding adequate support to the mother when test-weighing is ceased, as more of these mothers ceased exclusive breastfeeding at an early stage after discharge.

## Background

Breast milk is regarded to be the best nutrition for preterm infants [1]. However, preterm infants are often not strong enough to be exclusively breastfed in the first period of their lives. Therefore the mother is encouraged to start breast milk expression in order to feed (or supplement) the infant with expressed breast milk via a feeding tube until such time as it is possible to establish exclusive breastfeeding [2]. Even though the members of staff in the Neonatal Intensive Care Units (NICUs) put great effort into promoting breastfeeding, breastfeeding rates in Danish preterm infants are significantly lower (65%) at discharge than breastfeeding initiation rates in infants born at term (99%) [3],[4].

The lower breastfeeding rates in preterm infants might partly be explained by factors associated with preterm birth such as a lower gestational age (GA), multiple births [5], [6], [7], and maternal factors like smoking and low socio-economic status (SES) [3], [7], [8], [9], [10]. These factors are all shown to be negatively related to breastfeeding and are, together with previous breastfeeding experience, circumstances that are known at birth. After delivery a number of interventions are performed to facilitate breastfeeding. These are only partly based on evidence and there are significant differences between the neonatal departments, even in a small country like Denmark, with regards to clinical practices for the facilitation of breastfeeding.

### Clinical practices to facilitate breastfeeding

A lack of breast milk is one of the greatest barriers for establishing breastfeeding in preterm infants [11]. Mothers are recommended to initiate breast milk expression soon after the delivery but there is no consensus as to how early this should be done. In some studies it is recommended to start within the first 6 hours after delivery [12] whereas others suggest 24 hours [13]. A recent randomised controlled trial found that the initiation of breast milk expression within one hour, compared to six hours, after delivery doubled the mothers’ volume of milk for the first three weeks [14].

Skin-to-skin contact between the preterm infant and the mother/parents is also shown to promote breastfeeding [15], [16], [17], [18]. However, the effect of the timing of intermittent skin-to-skin contact has not been investigated. Rooming-in of the mother together with the infant in maternity units has shown to be associated with better breastfeeding outcomes [19], but research on the effect, on breastfeeding, of the admittance of mothers with their infants to NICUs, is sparse [20].

When the preterm infant is ready to initiate breastfeeding the use of a nipple shield is a possibility. In general nipple shields are not recommended for term infants [21], but reports on the advantages of nipple shield use for preterm infants came 13–17 years ago [22], [23] and, against this background, nipple shields have been widely recommended for preterm infants [24], [25]. A recent literature review concludes that current published research does not provide evidence for the safety or effectiveness of contemporary nipple shield use for either preterm or non-preterm infants [26].

Measurement of milk intake by weighing the infant immediately before and after a breastfeeding session – called test-weighing – has been recommended for preterm infants [24], [27], [28] and half of the Danish Neonatal Intensive Care Units (NICUs) use the test-weighing procedure by routine [2].

The use of a pacifier in preterm infants has been shown to relieve pain and reduce stress in the absence of the mother [29], [30], and is therefore widely used for preterm infants, although the use of a pacifier is not recommended for healthy newborns [21] as it is associated with lower breastfeeding outcomes [31]. An Australian randomised controlled trial of pacifier use in preterm infants showed no significant difference between groups in breastfeeding rates at discharge nor in breastfeeding duration [32], and a Brazilian study found that no use of a pacifier improved the likelihood of exclusive breastfeeding for preterm infants at six months with a 1,7 factor [33].

## Aim and Objectives

Our primary aim was to investigate the association between early breast milk expression, early initiation of skin-to-skin contact, rooming-in, nipple shield use, test-weighing, and pacifier use, and the establishment of exclusive breastfeeding at discharge, as well as at a predefined interval after discharge, in order to gain more evidence on which to base guidelines for mothers to preterm infants in the NICU.

## Materials and Methods

### Ethics Statement

The study was conducted in accordance with the Declaration of Helsinki [34] and approved by the Danish Data Protection Agency (j.nr. 2009-41-4024); surveys do not, by Danish law, need to be approved by the Biomedical Research Ethics Committee.

The mothers of the preterm infants gave written informed consent for participation.

### Design

The study was part of a prospective survey of a national Danish cohort of preterm infants based on questionnaires and structured telephone interviews conducted from September 2009 to December 2011.

### Setting

Denmark, with its 5.5 million inhabitants, has about 63,000 births per year, seven per cent of which are premature births. Denmark has public health care and all citizens can be treated in public hospitals free of charge. In connection with the birth, parents in Denmark are entitled to paid parental leave. Mothers have paid leave for a minimum of four weeks before delivery and up to 10.5 months after delivery, of which 7.5 can be shared with the father/partner. If the infant stays in hospital due to illness and/or prematurity, the leave is extended with the length of the hospital stay by a maximum of three months. Partners have two weeks’ leave following birth. An extra year of parental leave is possible without payment [35].

Except for many of the late preterm infants (GA 35 – 36 weeks), who do not need neonatal care, most of the preterm infants are admitted to one of Denmarks 19 NICUs, where they are hospitalised until breastfeeding is established (or exclusive breastfeeding is given up, and mixed feeding or bottle feeding is established) [2].

One of the NICUs provides low intensive care, 14 provide medium intensive care, and four provide high intensive care [2].

### Instruments

Based on a review of the literature and a National Expert Panel, three study-specific questionnaires were developed to be answered by the mother. The expert panel consisted of eight neonatal nurses, with 10–20 years each of experience in the breastfeeding of preterm infants. Four of them were International Board Certified Lactation Consultants (IBCLCs); three of them had research knowledge.

Questionnaire 1 (Q1) contained 38 questions including demographic questions about mother and infant, and questions about breastfeeding plans, experiences and self-efficacy, initiation of breast milk expression and skin-to-skin contact (Questionnaire S1).

Questionnaire 2 (Q2) contained 59 questions about breastfeeding initiation and establishment, feeding method(s) at discharge, breast milk expression, test-weighing, and the mother’s perceived support. There were also questions about the reasons for and timing of the use of pacifiers, bottle-feeding and nipple shields (Questionnaire S2).

Questionnaire 3 (Q3) contained 17 questions about the length of exclusive, and any, breastfeeding, possible reasons for ceasing, length of nipple shield use and breast milk expression, and perceived breastfeeding problems (Questionnaire S3).

In total the questionnaires included 30 interval scale variables, 76 categorical variables and three open questions.

Q1 was answered by the mothers approximately one week after delivery, and Q2 was answered by the mothers at the time of the infant’s discharge from NICU to home. Q3 was used for structured telephone interviews at 1, 4, 6 and 12 months of corrected age or until breastfeeding ceased, whichever occurred first.

### *Pilot study*

The three questionnaires were revised by two senior academic experts and, thereafter, 21 mothers from five different Danish NICUs tested the questionnaires for content and face validity. The pilot study led to minor changes.

### *Participants and datacollection*

All preterm infants, that is infants less than 37 gestational weeks old [36], who were admitted to the participating departments from 1 September 2009 to 31 August 2010 in their first five days of life, could participate in the study.

Infants were excluded if an interpreter was not available for the mother, if they were discharged to maternity units before five days of age, or if they died.

All departments in Denmark that, as a routine, take care of preterm infants during breastfeeding establishment were invited to participate in the study, which included 18 of the 19 NICUs, two special care units and one childrens’ department. All 21 units agreed to participation and, of these, 18 units adhered to the project protocol. The factors leading to non-adherence to the protocol were that less than two third of the eligible infants were approached or that enrolment slowed down during the last months. Telephone interviews and data entering were performed by the units and the National Expert Panel in charge of the study. The first author checked all the data and crosschecked the extreme and misleading data with the original responses.

The data from the survey will be publicly available on request, when all data has been analysed in the summer of 2015 and the data has been transferred to the Danish Data Archive.

### Outcomes

1) Exclusive breastfeeding at discharge was defined as the infant feeding directly at and from the breast, and it was thereby not equal to feeding the infant with breast milk from a bottle or other device. Exclusive breastfeeding at discharge could include medication and vitamins and, for a few infants, powder fortification mixed with the mother’s expressed milk (which for this study was considered as medication), but not water, formula or anything else. 2) Adequate duration of exclusive breastfeeding: The Danish Health Board’s recommendation for exclusive breastfeeding in preterm infants was chosen as the outcome for adequate duration, that is exclusive breastfeeding for four months plus half of the period of time the infant was born before estimated date of delivery [37]. For the regression analyses we used failure of exclusive breastfeeding and inadequate duration of exclusive breastfeeding so that an odds ratio (OR) lower than one would present factors with a positive association to breastfeeding, and a higher OR would present factors with a negative association to breastfeeding, since the majority of Danish preterm infants are breastfed [3].

### Variables and predictive variables

In the logistic regression analyses both variables concerning the infant and the mother that were known at birth and that were expected to have associations with breastfeeding, and variables reflecting the clinical practices that were used to establish or facilitate breastfeeding were entered. The variables were GA, in weeks, categorised in four groups: extremely preterm infants GA 24–27, very preterm infants GA 28–31, moderate preterm infants GA 32–34, and late preterm infants GA 35–36; multiple births; being small for gestational age (SGA) (defined as birth weight more than two standard deviations (SD) smaller than expected according to GA); the educational level of the mother (based on years of school and education), categorised in three groups: high (more than 16 years), intermediate (14–16 years), and low (less than 14 years); experience with breastfeeding, categorised into five groups: first time mothers, mothers who had not breastfed previous infants, previous breastfeeding of an infant exclusively for less than a month, for 1 – 4 months, and for more than four months; maternal smoking; and mode of delivery (Caesarean section). The variables reflecting clinical practices were the initiation of breast milk expression after delivery, categorised in five groups: before six hours, 6–12 hours, 12–24 hours, 24–48 hours, and later than 48 hours; first skin-to-skin contact before six hours postpartum; admitting mother and infant together into the NICU; nipple shield use during hospitalisation; test-weighing the infant at most breastfeeding sessions during transition from tube-feeding to breastfeeding; pacifier use during hospitalisation, categorised in three groups: no use of a pacifier, minimizing the use of a pacifier during breastfeeding transition, and the unrestricted use of a pacifier.

One or more of the following practices could be regarded as minimizing the use of a pacifier during the transition from tube-feeding to breastfeeding: predominantly using the pacifier during tube-feedings, painful or stressful events, predominantly using the pacifier in the mother’s absence, or removing the pacifier completely.

## Statistical Analyses

SPSS version 21.0 was used for statistical analyses. Failure of exclusive breastfeeding at discharge was analysed by means of logistic regression models, first the explanatory variables were analysed in univariate models and second the explanatory variables with a p-value of less than 0.1 were analysed simultaneously in a multiple model. Logistic regression analyses were performed with one infant per mother (to ensure that mothers of twins did not count as double [38]; for multiple births, the first born infant was included). The variables from the multivariate model analysing failure of exclusive breastfeeding at discharge were also used for analysing the associations with the second outcome, that is inadequate duration of exclusive breastfeeding, to see if associations persisted.

Descriptive statistics were used to present characteristics. The normally distributed results are reported with mean and standard deviation (SD); the remaining results are reported with median and interquartile range (IQR) or percentages [38]. Pearson’s Chi-Square test was used to determine statistically significant differences for nominal data. Values of p<0.05 were considered statistically significant.

## Results

### Participant selection

**Figure 1:**
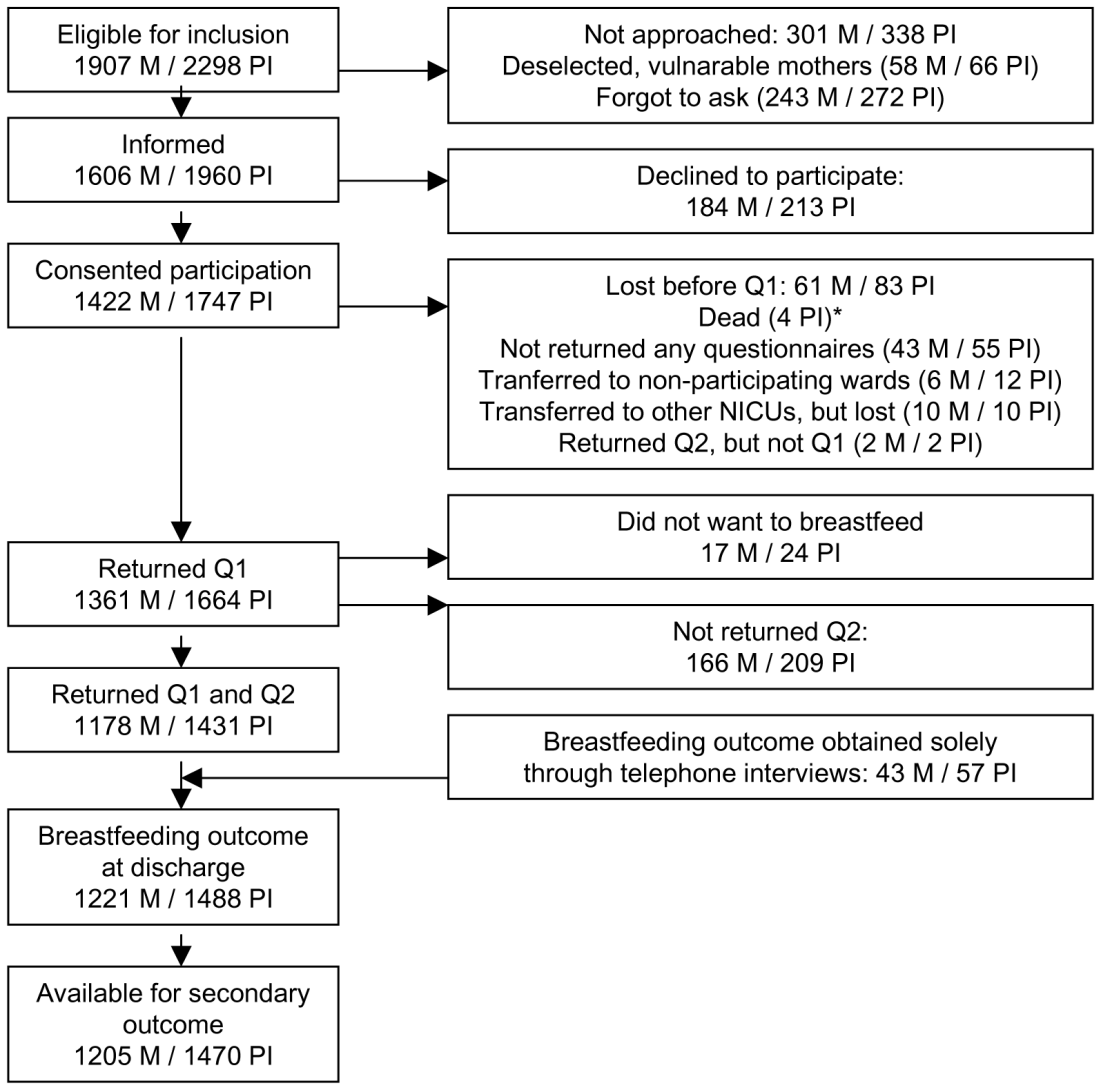
Flow chart. BF  =  breastfeeding, M  =  mothers, NICU  =  Neonatal Intensive Care Unit, PI  =  preterm infants, Q1  =  Questionnaire 1, Q2  =  Questionnaire 2. *The four infants who died after inclusion were all twins, no mothers were lost due to infant death.

Selections of participants are described in the flow chart (Figure 1). During the one year period 2,579 preterm infants were admitted to the NICUs; of these 281 were excluded either because an interpreter was not available for the mother (n  =  42), the infants were discharged to maternity units before five days of age (n  =  188), or had died (n  =  51). Thus 2,298 infants were eligible for inclusion. Of these, 1,664 infants participated with Q1, and 1,431 infants participated with both Q1 and Q2; an additional 57 infants with Q1 but without Q2 were also approached through structured telephone interviews, and primary and secondary outcomes were obtained. There were 1,488 infants (65% of eligible) available for primary outcome, 46% (n  =  60/131) of the extremely preterm infants, 65% of (n  =  257/398) the very preterm infants, 70% (n  = 688/984) of the moderate preterm infants, and 62% (n  =  483/785) of the late preterm infants (Table 1). For the secondary outcome, 1,470 infants were available, and the remaining 18 infants were included in the analysis as “not fulfilling breastfeeding duration”.

**Table 1:** Gestational age groups and drop out.

Gestational age, weeks		23 – 27	28 – 31	32 – 34	35 – 36
	N (%)	n (%)	n (%)	n (%)	n (%)
Eligible for inclusion	2298 (100)	131 (100)	398 (100)	984 (100)	785 (100)
Consented participation	1747 (76)	91 (69)	320 (80)	789 (80)	547 (70)
Breastfeeding outcomes	1488 (65)	60 (46)	257 (65)	688 (70)	483 (62)

N  =  total number, n  =  sub group numbers.

Of the participating infants 322 were transferred between the neonatal units. The number of participating mothers was 1,221.

Significantly fewer of the extremely preterm infants eligible for inclusion participated at discharge from NICU (p < 0.0001). Of mothers participating with Q1, significantly more of those who did not return Q2 had a lower level of education (p < 0.001).

### Participant characteristics

The mothers had a mean age of 31 years (SD  =  5), 93% were of Danish/Scandinavian origin, 98% had planned to breastfeed, 96% lived with the infant’s father and 97% of the mothers reported that their breastfeeding plans were supported by their partner. Twenty-two per cent of the mothers had multiple births (more twins than triplets), 36% of the infants were multiples, almost all (98%) of the infants had had skin-to-skin contact with the mother within the first week, and 99% of the infants had initiated breastfeeding. Table 2 shows rates of different characteristics in exclusive and non-exclusive breastfeeding groups.

**Table 2:** Proportions of infant and mother characteristics and clinical practices in exclusive and non-exclusive breastfeeding groups.

		Exclusive breastfeeding at discharge		
(One infant per mother)	Total n/N	Yes %	No %	
Multiple births	263/1221	18	31	****
SGA	191/1211	14	21	*
Boy	644/1221	51	58	*
Maternal education				NS
*High*	403/1207	32	37	
*Intermediate*	567/1207	47	48	
*Low*	237/1207	21	16	
Breastfeeding experience, lenght of excl. BF				****
*> 4 months*	196/1171	19	10	
*1* – *4 months*	167/1171	14	15	
*< 1 month*	21/1171	1	3	
*Not breastfed previous infants*	27/1171	1	5	
*First-time mothers*	760/1171	64	67	
Maternal smoking	123/1210	8	15	***
Mode of delivery, caesarean section	614/1219	48	57	**
Mother admitted together with infant to the NICU	344/1207	31	22	**
First breast milk expression				**
*< 6 hours pp*	255/1183	23	19	
*6* – *12 hours pp*	469/1183	41	38	
*12* – *24 hours pp*	288/1183	25	24	
*24* – *48 hours pp*	141/1183	11	15	
*> 48 hours pp*	30/1183	2	5	
Been skin-to-skin with mother within 6 hours pp	700/1217	61	48	****
Nipple shield use	629/1160	49	66	****
Pacifier use				****
*No pacifier*	132/1144	13	7	
*Minimizing use of a pacifier*	337/1144	34	19	
*Unrestricted use*	675/1144	53	73	
Test-weighing at most breastfeedings	351/1160	32	25	*

*  =  p < 0,05, **  =  p< 0,01, ***  =  p< 0,001, ****  =  p< 0,0001.

BF  =  breastfeeding, excl.  =  exclusive, n/N  =  number of infants or mothers with the characteristic/number of responses, NS  =  Non-significant, pp  =  postpartum, SGA  =  Small for gestational age.

### Breastfeeding rates

At discharge, 68% of the infants were exclusively breastfed, 17% were partially breastfed, and 15% were not breastfed. Some of the infants were fed with breast milk from bottles. Adding those infants to the breastfed infants, 77% were exclusively breast milk fed at discharge, 15% were partially breast milk fed, and eight per cent were not fed breast milk at all at the time of discharge. Thirty-one per cent were breastfed exclusively for the recommended duration.

The exclusive breastfeeding rates at discharge varied significantly between the participating units from 53 to 83% (p < 0.0001), as did rates of nipple shield use from 35 to 67% (p < 0.0001), pacifier use from 60 to 100% (p < 0.0001), and the use of test-weighing from 0 to 87%(p > 0.0001). Counting one infant per mother, 76% of the infants who were test-weighed at most breastfeeding sessions were exclusively breastfed at discharge compared to 69% of the infants who were not test-weighed at most breastfeeding sessions (p  =  0.02). One month after discharge the corresponding proportion was 59% and 57%, respectively, with no statistically significant difference.

### Breastfeeding at discharge

The univariate analyses showed that all the factors from the set of variables, together with the gender of the infant, were significantly associated with exclusive breastfeeding at discharge (Table 3). The multivariate analysis showed that the following characteristics were each independently associated with significantly higher odds of failure of exclusive breastfeeding at discharge: extremely preterm and very preterm infants, multiple births (twins and triplets) and boys. Furthermore, infants to mothers who had not breastfed previous infants or who smoked had higher odds for failure of exclusive breastfeeding at discharge (OR 5.6 (95% CI 2.0 – 15.9), and 2.2 (95% CI 1.4 – 3.7) respectively).

**Table 3:** Odds for failure of exclusive breastfeeding at discharge from NICU to home.

(one infant per mother)						
		*Unadjusted analyses*			*Adjusted analysis*	
					*(N = 1007)*	
*Infant and mother characteristics*	N	Prev.	OR (95% CI)		OR (95% CI)	
Gestational age groups, GA 24 – 27 weeks	1221	4%	3.0 (1.7 – 5.5)	***	2.9 (1.3 – 6.4)	**
GA 28 – 31 weeks		18%	1.6 (1.1 – 2.3)	*	1.8 (1.1 – 2.9)	*
GA 32 – 34 weeks		48%	1.0 (0.8 – 1.3)		1.0 (0.7 – 1.4)	
GA 35 – 36 weeks (ref)		33%	1		1	
Multiple birth	1221	22%	2.1 (1.6 – 2.9)	****	2.0 (1.4 – 2.9)	***
Small for gestational age	1211	16%	1.7 (1.2 – 2.3)	**	1.2 (0.8 – 1.9)	
Gender, boys	1221	53%	1.4 (1.1 – 1.7)	*	1.7 (1.3 – 2.3)	**
Maternal education, high ( ref)	1207	20%	1		1	
Intermediate		47%	1.4 (1.0 – 2.0)		1.3 (0.9 – 2.0)	
Low		33%	1.5 (1.1 – 2.2)	*	1.2 (0.8 – 1.9)	
Breastfeeding experience, lenght of excl. BF	1171					
>4 mo. (ref)		17%	1		1	
1 – 4 months		14%	2.0 (1.2 – 3.3)	**	1.6 (0.9 – 2.9)	
< 1 month		2%	4.3 (1.7 – 11.0)	**	2.7 (0.9 – 8.5)	
Not breastfed previous infants		2%	8.1 (3.4 – 19.2)	****	5.6 (2.0 – 15.9)	**
First time mothers		65%	2.0 (1.4 – 3.0)	**	1.4 (0.9 – 2.3)	
Maternal smoking	1210	10%	1.9 (1.3 – 2.8)	**	2.2 (1.4 – 3.7)	**
Mode of delivery, caesarean section	1219	50%	1.4 (1.1 – 1.8)	**	1.1 (0.8 – 1.5)	
***Breastfeeding practices***						
Mother admitted together with infant to the NICU	1207	29%	0.6 (0.5 – 0.9)	**	0.8 (0.6 – 1.2)	
First breast milk expression, < 6 hours pp (ref)	1183	22%	1		1	
6 – 12 hours pp		40%	1.1 (0.8 – 1.6)		1.0 (0.7 – 1.6)	
12 – 24 hours pp		24%	1.2 (0.8 – 1.8)		1.1 (0.7 – 1.8)	
24 – 48 hours pp		12%	1.7 (1.1 – 2.7)	*	1.5 (0.8 – 2.6)	
> 48 hours pp		3%	3.8 (1.8 – 8.3)	**	4.9 (1.9 – 12.6)	**
Been skin-to-skin with mother within 6 hours pp	1217	58%	0.6 (0.5 – 0.8)	****	1.1 (0.8 – 1.6)	
Nipple shield use	1160	54%	2.0 (1.5 – 2.6)	****	2.3 (1.6 – 3.2)	****
Pacifier use, no pacifier	1144	12%	0.4 (0.2 – 0.6)	***	0.6 (0.3 – 1.0)	*
Minimizing use of a pacifier		30%	0.4 (0.3 – 0.6)	****	0.4 (0.3 – 0.6)	****
Unrestricted use of a pacifier (ref)		59%	1		1	
Test-weighing at most breastfeeds	1160	30%	0.7 (0.5 – 0.9)	*	0.6 (0.4 – 0.8)	**

*  =  p < 0,05, **  =  p< 0,01, ***  =  p< 0,001, ****  =  p< 0,0001.

BF  =  breastfeeding, CI  =  confidence interval, GA  =  Gestational age, **N**  =  Number included in unadjusted analyses, (N  =  1007)  =  Number included in adjusted analysis NICU  =  Neonatal Intensive Care Unit, OR  = odds ratio, pp  = post partum, prev  =  prevalence.

**Figure 2:**
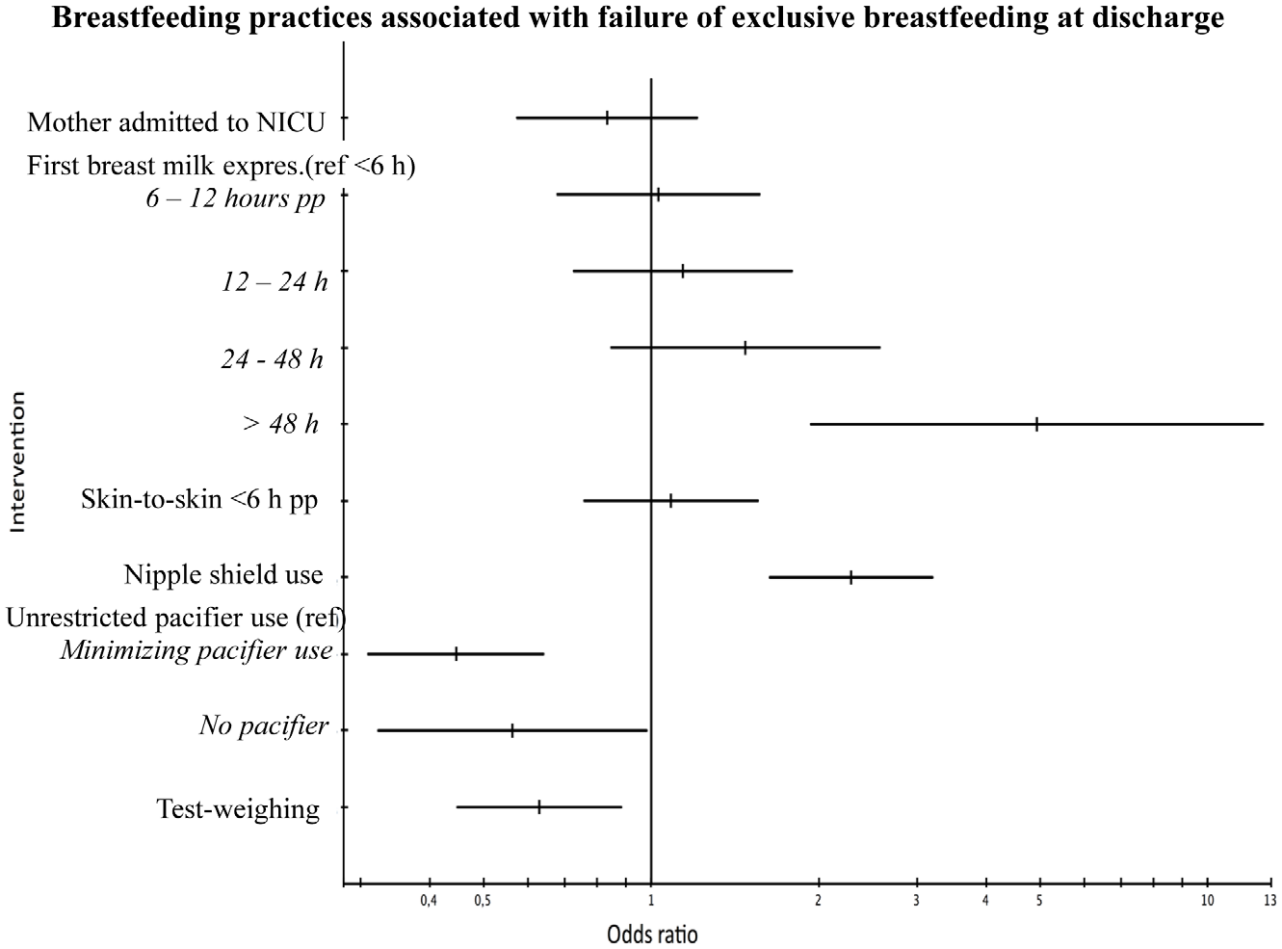
Forest plot. BM expres.  =  Breast milk expression, h  =  hours, NICU  =  Neonatal Intensive Care Unit, pp  =  postpartum, ref  =  reference.

A number of clinical practices were associated with a significantly higher OR of failure of exclusive breastfeeding at discharge (Figure 2). Infants using nipple shields had a 2.3-fold increased risk. Delayed initiation of breast milk expression showed a dose-response effect: the later the initiation, the higher the risk for failure to exclusively breastfeed at discharge, although only initiation later than 48 hours postpartum reached significance (OR 4.9 (95% CI 1.9 – 12.6)). Test-weighing the infant at most breastfeeding sessions during transition from tube-feeding to breastfeeding was associated with a lower risk (OR 0.6 (95% CI 0.4 – 0.8)) of failure to exclusively breastfeed at discharge and thus positively related to exclusive breastfeeding. The same association was seen for no use of a pacifier and for minimizing the use of a pacifier during breastfeeding transition OR 0.6 (95% CI 0.3 – 1.0) and 0.4 (95% CI 0.3 – 0.6) respectively.

The difference in exclusive breastfeeding rates between the NICUs persisted when adjusting for infant and maternal characteristics and clinical procedures (p  =  0.001).

### Duration of breastfeeding

Testing factors associated with adequate duration of exclusive breastfeeding showed that multiple births and maternal smoking had similar results with regards to exclusive breastfeeding at discharge (Table 4). Neither gestational age groups nor the infant’s gender were associated with adequate duration of exclusive breastfeeding.

**Table 4:** Odds for inadequate duration of exclusive breastfeeding.

(one infant per mother)		
	*Adjusted analysis*	
	*(N = 1007)*	
***Infant and mother characteristics***	**OR (95% CI)**	
Gestational age groups, GA 24 – 27 weeks	1.6 (0.7 – 3.7)	
GA 28 – 31 weeks	1.4 (0.9 – 2.2)	
GA 32 – 34 weeks	1.2 (0.9 – 1.7)	
GA 35 – 36 weeks (ref)	1	
Multiple birth	2.4 (1.6 – 3.6)	****
Small for gestational age	1.2 (0.8 – 1.7)	
Gender, boys	1.1 (0.8 – 1.5)	
Maternal education, high ( ref)	1	
Intermediate	1.6 (1.1 – 2.3)	**
Low	2.6 (1.7 – 3.9)	****
Breastfeeding experience, lenght of excl. BF, >4 mo. (ref)	1	
1 – 4 months	3.5 (2.1 – 5.9)	****
< 1 month	11.1 (2.2 – 54.7)	**
Not breastfed previous infants	8.7 (2.3 – 32.2)	**
First time mothers	2.7 (1.8 – 4.0)	****
Maternal smoking	3.4 (1.8 – 6.5)	***
Mode of delivery, caesarean section	0.9 (0.7 – 1.3)	
***Breastfeeding practices***		
Mother admitted together with infant to the NICU	0.8 (0.6 – 1.1)	
First breast milk expression, < 6 hours pp (ref)	1	
6 – 12 hours pp	1.2 (0.8 – 1.7)	
12 – 24 hours pp	1.6 (1.0 – 2.4)	*
24 – 48 hours pp	1.8 (1.0 – 3.1)	*
> 48 hours pp	2.1 (0.8 – 5.6)	
Been skin-to-skin with mother within 6 hours pp	1.3 (0.9 – 1.8)	
Nipple shield use	1.4 (1.1 – 1.9)	*
Pacifier use, no pacifier	0.8 (0.5 – 1.3)	
Minimizing use of a pacifier	0.8 (0.6 – 1.1)	
Unrestricted use of a pacifier (ref)	1	
Test-weighing at most breastfeeds	0.9 (0.7 – 1.2)	

*  =  p < 0,05, **  =  p< 0,01, ***  =  p< 0,001, ****  =  p< 0,0001.

BF  =  breastfeeding, CI  =  confidence interval, GA  =  Gestational age, (N  =  1007)  =  Number included in adjusted analysis NICU  =  Neonatal Intensive Care Unit, OR  = odds ratio, pp  = post partum.

In addition, maternal education at either an intermediate or low level was a risk factor. Compared to mothers who had breastfed a previous infant for more than four months, less or no breastfeeding experience was associated with a higher OR for inadequate breastfeeding duration.

**Figure 3:**
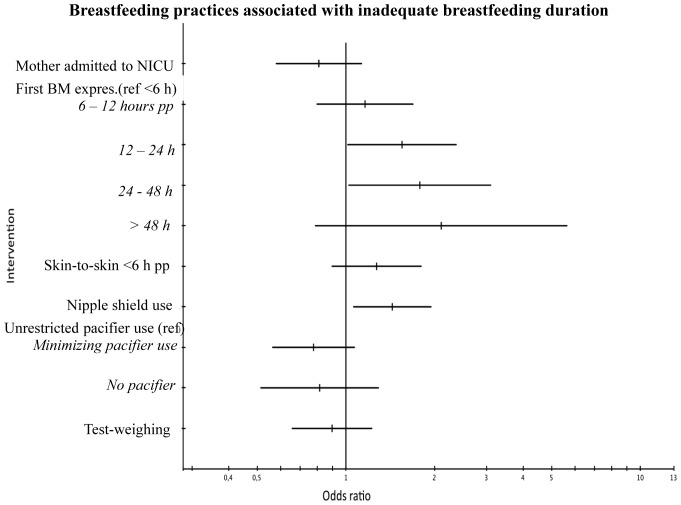
Forest plot. BM expres.  =  Breast milk expression, h  =  hours, NICU  =  Neonatal Intensive Care Unit, pp  =  postpartum, ref  =  reference.

As for clinical practices, the delayed initiation of breast milk expression again showed a dose-response effect – the later the expression was initiated the higher the OR –with initiation 12 – 24 hours and 24 – 48 hours postpartum reaching significance for inadequate breastfeeding duration (OR 1.6 (95% CI 1.0 – 2.4) and 1.8 (95% CI 1.0 – 3.1), respectively) (Figure 3). Also, the use of nipple shields was associated with a higher OR for inadequate breastfeeding duration (OR 1.4 (95% CI 1.1 – 1.9)). The use of test-weighing or the use of a pacifier were not significantly associated with adequate breastfeeding duration.

## Discussion

We presented data from a national cohort of preterm infants and found a relatively high rate of exclusive breastfeeding at discharge (68%) compared to 27 – 60% of preterm infants in US and Scandinavian studies [3], [39], [40], [41], showing that it is possible to establish exclusive breastfeeding in the majority of preterm infants. The odds of breastfeeding failure at discharge were inversely related to gestational age: the lower the gestational age the higher the odds for breastfeeding failure at discharge, even when we corrected for potential confounders such as multiple births, mode of delivery, SES, and maternal smoking. The association is not surprising and could be explained by an increased risk of morbidity and a longer time where the infant is admitted to the NICU. However, our data also showed that if the mother were to succeed in the establishment of exclusive breastfeeding at discharge the significant impact of gestational age disappeared when the duration of exclusive breastfeeding was the outcome. The mother’s education is usually associated with breastfeeding establishment and duration [3], [7], [9], [10], [42] but in our study the association was only seen in connection to breastfeeding duration. This was a surprising finding and could be due to the NICUs’ ability to support less educated mothers during hospitalisation.

The present study is the first to examine several different time intervals of initiation of breast milk expression, and showed a dose-response effect on both outcomes: the later the initiation the higher the odds – increasing from 1.0 to 4.9 – for breastfeeding failure at discharge and for inadequate breastfeeding duration. The 12–24 hours and 24–48 hours intervals were significantly associated with breastfeeding duration (p<0,05). For breastfeeding failure at discharge we found a more significant association (p<0,01), but with a later time interval (> 48 hours postpartum). The results indicate that exclusive breastfeeding is not impossible if breast milk expression is initiated later than 12 hours postpartum, but it seems that initiating this even before six hours postpartum would be helpful for mothers who want to breastfeed their preterm infant. These results support other studies finding that breast milk expression should be initiated at an early stage after the delivery [12], [13], [14]. From another study we know that 89% of the Danish NICUs would advise the mothers to initiate breast milk expression within six hours after delivery [2], but only 22% of the mothers did so. Even though our results could be biased by other factors such as the mother’s disease, we consider it reasonable to recommend that breast milk expression should be initiated as soon as possible.

The timing of initiation of skin-to-skin contact and the admittance of the mother directly after delivery together with the infant to the NICU did not show a significant association to breastfeeding in the present study. Our study was not designed to give definitive answers to whether or not these practices promote breastfeeding and, furthermore, it should be emphasized that there may be other benefits of these practices for the mother-infant relationship than that of promoting breastfeeding. Skin-to-skin contact has previously been positively associated with breastfeeding preterm and non-preterm infants [15], [16], [17], [43], but only one study of preterm infants demonstrated that the initiation of continuous skin-to-skin contact before 24 hours postpartum was positively associated with exclusive breastfeeding at six months of age [19]. Most of the infants (98%) in the present study had skin-to-skin contact during the NICU stay, and it seems that even if 42% of the mothers were not able to have early skin-to-skin contact with their preterm infants this was not an essential barrier to the establishment of breastfeeding. On the other hand, it has previously been shown that it is possible to establish skin-to-skin contact even with extremely preterm infants [44], and although there is no evidence that this promotes breastfeeding we find no reason not to recommend this practice unless there are medical reasons that oppose this.

We did not ask the 29% of mothers in the present study who were admitted to the NICU directly after delivery together with the infant, where in the NICU they had slept (next to the infant, or in another room in the NICU), and we did not ask for how long they had stayed in the NICU. Mothers, who were not admitted to the NICU directly after delivery, were not asked if they were admitted to the NICU later or for how long they could sleep in the NICU. From a previous study we know that all Danish NICUs offered the mothers rooming-in some days before discharge, and in 42% of the NICUs the mother could have a bed in the NICU when she was discharged from maternity ward [2]. Therefore, we still lack knowledge of whether or not rooming-in for the infant’s whole hospitalisation period (defined as sleeping together) is associated with breastfeeding success for preterm infants, just as it is for non-preterm infants [19]. A Swedish study found that mothers separated from their newborn infants experienced emotional strain and anxiety; they felt like they were outsiders, and experienced a lack of control when the infant was admitted to neonatal intensive care [45]. A Danish study found that the possibility for rooming-in in a neonatal ward could help the parents feel like a family and not just visitors to their own baby [46]. For these reasons efforts should still be made to avoid separating the mother and the preterm infant.

The use of a nipple shield has previously been described as a facilitator of breastfeeding in preterm infants, but only in small studies with 15 and 34 infants and no control groups [22], [23]. In our study the use of nipple shields was negatively associated with exclusive breastfeeding establishment and duration. Nipple shields are often used to solve breastfeeding problems, some of the problems may be hard to solve, even with a nipple shield, but the huge variation in nipple shield use between the NICUs hospitalising similar infants (35–67% of discharged infants) indicates that a nipple shield was not always used because of severe latching problems. Thus, although our results could be biased, they suggest that the use of nipple shields does not promote breastfeeding in preterm infants, just as it does not promote breastfeeding in non-preterm infants [26].

Test-weighing the infant at most breastfeeding sessions during transition was, in the present study, protective to exclusive breastfeeding at discharge, but had no association with breastfeeding duration. Mothers of preterm infants are concerned about their small infants getting enough milk and growing well, which could be a reason why test-weighing seems to help establish exclusive breastfeeding. The protective effect was eliminated within one month of discharge, indicating that mothers using test-weighing in the NICU ceased exclusive breastfeeding earlier after discharge, when they could not measure the amount of milk that their infant sucked. Earlier studies from Sweden show contadictory results: One Swedish study comparing two units, found no differences in breastfeeding outcome at discharge [47], whereas another Swedish study using a quasi-experimental design found that infants in the “not test-weighing group” were twice as likely to fail exclusive breastfeeding at discharge [48], as was the case in the present study. Test-weighing has previously been found to be associated with a shorter duration of exclusive breastfeeding for term infants [49].

Pacifier use is also controversial. The multivariate analysis showed that no use of a pacifier and the minimization of the use of a pacifier during breastfeeding transition were both positively associated with exclusive breastfeeding at discharge, but not associated with breastfeeding duration. A Brazilian study supports that not using a pacifier at all is positive for exclusive breastfeeding of preterm infants [33], as it is for term infants [31]. To our knowledge, minimizing the use of a pacifier during breastfeeding transition has not previously been studied for preterm infants, but could be a useful intervention for the many preterm infants using pacifiers during hospitalisation.

### Strengths and limitations

The strengths of the present study are: the large number of participating preterm infants and mothers, the fact that the study is national, and the carrying out of repeated structured telephone interviews so as to reduce recall bias.

It is a clear limitation that our observational study was not designed to establish cause and effect relationships. However, a large study like ours provides important evidence from the daily clinic that supports, or questions, the recommendations based on data from smaller studies. Another limitation is that two out of four high intensive neonatal units did not enrol mother-infant pairs, and although mostly all infants were transferred to participating NICUs, more of the infants with the lowest gestational age were not approached. That could be because the infants were more than a month old at transfer, which could be a barrier for asking the mothers to participate in a study that optimally should have begun a week after the infant’s birth. It is also known that participants with poorer health outcomes are more reluctant to participate in surveys and more often drop out from cohorts [50]. Also, the high drop-out rate of extremely preterm infants could indicate that they are at an even greater risk for not being exclusively breastfed than this study could show. The higher drop-out rate of less educated mothers before discharge, could affect the result of education having no association to exclusive breastfeeding at discharge.

The structured telephone interviews, conducted by NICU nurses experienced in breastfeeding, could serve as an intervention, where the mothers could get answers to their breastfeeding questions, as there were no limitations in the nurses’ answers to the mothers’ questions. Results in the present study of late preterm infants cannot be generalised to all late preterm infants, as late preterm infants admitted to NICUs have more health problems than late preterm infants cared for in maternity units. During the study period the Danish hospitals went through reductions in hospital staff influencing most of the participating units, resulting in some units not having the time for telephone interviews and entering data.

## Conclusions

Mothers of preterm infants should be guided to initiation of early breast milk expression before 12 hours postpartum, as this may result in better breastfeeding outcomes. Our data also suggest that the use of nipple shields should be restricted for preterm infants. Use of test-weighing and minimizing the use of a pacifier during breastfeeding establishment may promote exclusive breastfeeding, but more research is needed regarding adequate support to the mother when test-weighing is ceased, as more of these mothers ceased exclusive breastfeeding early after discharge. In order to increase exclusive breastfeeding rates for preterm infants, special breastfeeding support and guidance should be given to first-time mothers, smokers, mothers with a lower level of education, mothers of infants younger than 32 gestational weeks, and mothers of twins and triplets.

## Supporting Information

Questionnaire S1 Breastfeeding survey. Questionnaire 1 for the baby’s mother at the beginning of the baby’s hospitalization.
(PDF)

Questionnaire S2 Breastfeeding survey. Questionnaire 2 for the baby’s mother at the baby’s discharge.
(PDF)

Questionnaire S3 Breastfeeding survey. Questionnaire 3 used for telephone interviews with the baby’s mother.
(PDF)
