# Supplementary material for: Factors Associated with Exclusive Breastfeeding of Preterm Infants. Results from a Prospective National Cohort Study
Source: PLoS One. 2014 Feb 19;9(2):e89077. doi: 10.1371/journal.pone.0089077 (PMC3929624; doi:10.1371/journal.pone.0089077)
Supplement: Questionnaire S1 — Breastfeeding survey. Questionnaire 1 for the baby’s mother at the beginning of the baby’s hospitalization. (PDF) [file pone.0089077.s001.pdf]

Ward number _____   Code number _________

Breastfeeding study of preterm infants in neonatal wards in Denmark 2009 – 2011

Questionnaire 1 for the baby’s mother

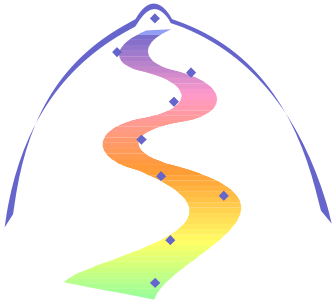

Beginning of hospitalization

Thank you for participating in the study.
We ask you to answer this questionnaire when your baby is about one week old. The questions are about you, the delivery, how your first week has passed and whether you have started breast milk pumping and/or are breastfeeding.

For each question, you are asked to either write your answer or tick a box.
If you may tick more than one box, this will be stated in the question.

You can always ask the nursing staff for help in completing the questionnaire if you need to. Once you have completed the questionnaire, please return it to the nursing staff.

After you have returned this form, you will receive the next questionnaire, which you are requested to complete and return when your baby is discharged from the neonatal ward.

The questionnaire is filled in; date: _________

The first questions are about your baby's birth (delivery) and how you and your baby are doing right now.

1. Your baby was born: date: ______________

2. The gestational age of your baby at birth: ______weeks _______days

3. Birth weight: ______________ grams

4. Your baby is:

   A girl ☐1

   A boy ☐2

5. Your baby is born by Caesarean section

   Yes ☐1

   No ☐2

6. Did you have complications in connection to labour/delivery, which prevented you, for more than the first 24 hours, from being together with your baby?

   Yes ☐1

   No ☐2

7. Have you and your baby been admitted to different wards after the delivery?

   Yes ☐1

   No ☐2

8. Today your baby is ________ days old.

9. Yesterday your baby was placed in:

   A closed incubator ☐1

   An open incubator ☐2

   A cot/bed ☐3

Questions about breastfeeding

The next questions are about your experiences and thoughts about breastfeeding.

10. Did you plan to breastfeed your baby?

    Yes (*Proceed to question **12***) ☐1

    No ☐2

11. What is your reason for not breastfeeding?

    *(Please answer this question and proceed to question **16**)*

    I do not want to breastfeed ☐1

    I cannot breastfeed (e.g. because of breast surgery) ☐2

    I am not allowed to breastfeed (e.g. because of medication) ☐3

    Other reasons ☐4

    Please describe: ____

12. For how long have you planned to breastfeed your baby?

    Until the baby is __________ month(s) old

13. For how long have you planned to breastfeed your baby if your baby was born at the estimated date of delivery?

    Until the baby is _______ month(s) old

14. Of how great importance is it to you to breastfeed?

    Very great importance ☐1

    Great importance ☐2

    Some importance ☐3

    Little importance ☐4

    No importance ☐5

    I don't know ☐6

15. How confident are you that you can breastfeed your preterm baby for as long as you have planned?

- Very confident ☐1
- Confident ☐2
- Don't know ☐3
- Uncertain ☐4
- Very uncertain ☐5

16. Does your partner support your choice of breastfeeding?

- Yes ☐1
- No ☐2
- I don't know ☐3
- I'm alone with my baby ☐4

17. Have you breastfed before?

- Yes ☐1
- No, this is my first child ☐2
  (Proceed *to question **19***)
- No, I haven't breastfed my other children ☐3
  (Proceed *to question **19)***

18. For how long have you breastfed your children?

Child No. 1: Exclusive breastfeeding for ______month(s)
Breastfeeding for a total of _______month(s)

Child No. 2: Exclusive breastfeeding for ______ month(s)
Breastfeeding for a total of ______ month(s)

Child No. 3: Exclusive breastfeeding for ______ month(s)
Breastfeeding for a total of _______month(s)

Child No. 4: Exclusive breastfeeding for _______month(s)
Breastfeeding for a total of _______ month(s)
(if you have more children, continue here)

19. What are your experiences with breastfeeding in your close family/network?

- Mostly positive experiences ☐1
- Mostly negative experiences ☐2
- No experiences ☐3

20. What are your experiences with breastfeeding in your partner's close family/network?

- Mostly positive experiences ☐1
- Mostly negative experiences ☐2
- No experiences ☐3

21. What are your experiences with breastfeeding preterm babies in your and your partner's close family/network

- Mostly positive experiences ☐1
- Mostly negative experiences ☐2
- No experiences ☐3

Questions about breast milk pumping

22. Have you started breast milk pumping for your baby?

- Yes ☐1
- No *(Proceed to question **26**)* ☐2

23. When did you pump for the first time?

- Before my baby was 6 hours old ☐1
- When my baby was 6 – 12 hours old ☐2
- When my baby was 12 – 24 hours old ☐3
- When my baby was 24 – 48 hours old ☐4
- When my baby was more than 48 hours old ☐5

24. How many times have you pumped for the last 24 hours? _________ times

25. How much milk did you pump in total for the last 24 hours?

- Less than 50 ml ☐1
- 50 – 200 ml ☐2
- 200 – 400 ml ☐3
- 400 – 750 ml ☐4
- More than 750 ml ☐5

26. How is your baby being fed right now? (*You may tick more than one box)*

- Breastfeeding ☐1
- Feeding tube ☐2
- Cup ☐3
- Lact-aid ☐4
- Finger-feeding ☐5
- Bottle ☐6
- Intravenous nutrition ☐7

Questions about skin-to-skin contact

(With skin-to-skin contact we mean that your baby is only dressed in a nappy, maybe a cap and socks, and maybe an open blouse, but in a way that your baby's stomach, chest and legs are in direct contact with your (or another adult's) bare chest.)

27. When did you (the mother) at first have your baby skin-to-skin?

- Immediately after the baby was born ☐1
- Short time after delivery = 0 – 6 hours ☐2
- 6 – 24 hours after delivery ☐3
- 1 – 2 days after delivery ☐4
- More than two days after delivery ☐5
- My baby has not been skin-to-skin with me ☐6

28. When did your partner (or another adult) at first have your baby skin-to-skin?

- Immediately after the baby was born ☐1
- Short time after delivery = 0 – 6 hours ☐2
- 6 – 24 hours after delivery ☐3
- 1 – 2 days after delivery ☐4
- More than two days after delivery ☐5
- My baby has not been skin-to-skin with my partner (or another adult) ☐6

29. For how long did your baby have skin-to-skin contact yesterday?
(You are supposed to add the hours, if your baby was skin-to-skin with persons other than yourself)

- 0 – 1 hours ☐1
- 1 – 2 hours ☐2
- 2 – 4 hours ☐3
- 4 – 6 hours ☐4
- 6 – 8 hours ☐5
- 8 – 12 hours ☐6
- More than 12 hours ☐7
- My baby did not have skin-to-skin contact yesterday ☐8

General questions about you and your schooling

30. How old are you? ____________ years

31. How do you live?

Together with my baby’s father	☐1
Together with an adult other than my baby’s father	☐2
Alone	☐3

32. Do you have other children at home (apart from your new-born baby)?

Yes	☐1
No	☐2

If yes, please give the number _____ and ages of the children ____________

33. In which country are you born? _________________

34. Which language do you speak at home? _________________

35. Which schooling have you completed?

9th grade (or lower) without examination	☐1
9th grade with examination	☐2
10th grade with examination	☐3
Senior high (10th – 12th grade)	☐4
Other	☐5

Please describe: ____

36. Which educational courses/programmes have you completed or are you taking?

None	☐1
Labour-market courses, special training programmes	☐2
Occupational programmes (apprenticeship, traineeship e.g. carpentry, welding)	☐3
Short secondary educational programmes (2-3 years)	☐4
Medium-length secondary educational programmes (3-4 years)	☐5
Long secondary educational programmes (4-6 years or longer)	☐6

37. How was your employment situation before delivery?

Student	☐1
Homemaker	☐2
Unemployed	☐3
Retired	☐4
Working part time	☐5
Working full time	☐6

38. Do you smoke?

Yes	☐1
No	☐2

Thank you for completing the questionnaire.

Please return the questionnaire to the staff.

The survey is done in cooperation with
Knowledge Centre for Breastfeeding Infants with Special Needs
Copenhagen University Hospital, Rigshospitalet
Department of Neonatology
Copenhagen
Denmark
