# Supplementary material for: Factors Associated with Exclusive Breastfeeding of Preterm Infants. Results from a Prospective National Cohort Study
Source: PLoS One. 2014 Feb 19;9(2):e89077. doi: 10.1371/journal.pone.0089077 (PMC3929624; doi:10.1371/journal.pone.0089077)
Supplement: Questionnaire S2 — Breastfeeding survey. Questionnaire 2 for the baby’s mother at the baby’s discharge. (PDF) [file pone.0089077.s002.pdf]

Ward number _____   Code number ________

Breastfeeding study of preterm infants in neonatal wards in Denmark 2009 – 2011

Questionnaire 2 for the baby's mother

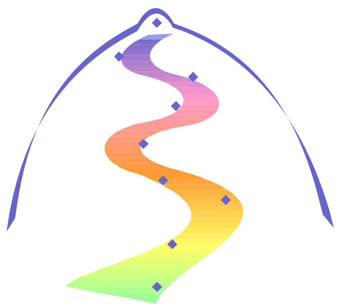

Discharge

This questionnaire is about how your baby has started oral feeding, and about your baby's hospital stay, as well as the breastfeeding support you have received.

You can complete the first 12 questions as your baby moves forward in oral feeding, and fill in the rest of the answers at discharge. However, even though you don't have to hand in the form until your baby is discharged, it's a good idea to read through it now already.

You are always welcome to ask the nursing staff for help in completing the questionnaire if needed.

Questions about your baby's first breastfeeding experience

When was your infant first put to the breast to lick, taste and perhaps latch on the breast? (Not necessarily sucking or sinking)

1. Date: ___________
2. Weight this day: ____________ (approx.)
3. At the same time did your infant then get: *(Please answer all 3 questions with a tick in a box on each line)*

	Yes	No
a. Nasal CPAP?	☐1	☐2
b. Oxygenation?	☐1	☐2
c. Break from nasal CPAP or oxygen?	☐1	☐2

Questions about the period when your baby begins to take oral feeds

When did your baby at first complete an oral feeding?

4. Date: __________
5. Weight this day: ____________ (approx.)
6. In what way was your baby fed at this feeding?
(You may tick more than one box)

Breastfed	☐1
Bottle-fed	☐2
Cup-fed	☐3
With Supplemental nursing system/Lact-aid	☐4
Other	☐5

Please describe: ______________________________________

7. In what ways was your baby fed at other feedings for this particular day?
(You may tick more than one box)

Breastfed	☐1
Tube-fed	☐2
Bottle-fed	☐3
Cup-fed	☐4
With Supplemental nursing system/Lact-aid	☐5
Other	☐6

Please describe: ______________________________________

When did your baby take all feeding orally without tube-feeding?

8. Date: ___________
9. Weight this day: ____________ (approx.)
10. How did your baby feed this day?

My baby was only breastfed	☐1
My baby was both breastfed and bottle-fed	☐2
My baby was only bottle-fed	☐3
Other:	☐4

Please describe: ______________________________________

11. Did your baby need tube-feeding again later on?

Yes	☐1
No *(Proceed to question **13**)*	☐2

12. If yes, since what date has your baby been completely without tube-feedings?

Since date____________

Questions about how your baby is fed at the final discharge from hospital

13. When was your baby discharged from hospital? Date: ___________

14. Your baby's weight at the final discharge: _____________ grams (approx.)

15. How is your baby fed at the final discharge?

My baby is only breastfed	☐1
My baby is both breastfed and bottle-fed	☐2
My baby is only bottle-fed *(go to question **20**)*	☐3
Other<br>Please describe: ____	☐4

16. For how long do you plan to continue breastfeeding?

Until my infant is approx. _______ months (counting from the baby's date of birth)

17. How confident are you that you can breastfeed your preterm baby for as long as you have planned?

Very confident	☐1
Confident	☐2
Don't know	☐3
Uncertain	☐4
Very uncertain	☐5

18. How do you experience breastfeeding today and yesterday (the day your baby is discharged from hospital and the day before that)?

Unproblematic	☐1
Slightly problematic	☐2
Problematic	☐3
Very problematic	☐4

19. If you experience breastfeeding as being problematic in any way, please describe what makes it this way:

____
____
____
____

You only need to answer question 20 – 23 if your baby is receiving milk in a way other than by breastfeeding.

20. What does your baby get from the bottle?

Expressed breast milk	☐1
Infant formula	☐2
Both expressed breast milk and formula	☐3

21. What is the reason that your baby is not fully breastfed at discharge?
(You may tick more than one box)

I did not have enough milk	☐1
I've stopped because of wounds, cracks or sore nipples	☐2
I've stopped because of mastitis	☐3
I have inverted/flat nipples	☐4
My baby could not/would not breastfeed	☐5
My baby was not getting satisfied by breastfeeding	☐6
My baby was not gaining enough weight by breastfeeding	☐7
I need to know exactly how much my baby gets	☐8
I feel bound to breastfeeding	☐9
Other<br>Please describe: ____	☐10

22. When was the decision made that your baby should not be fully breastfed?
Date: __________ (approx.)

23. Did you get support from the staff when you could not fully breastfeed or stopped expressing breast milk?

Yes	☐1
No	☐2

Questions about the use of a nipple shield and pacifier for your premature baby

24. Have you used a nipple shield to breastfeed your premature baby?
    - Yes ☐1
    - No *(proceed to question **28**)* ☐2

25. What was/were the reason/reasons that you used a nipple shield?
    (You may tick more than one box)
    - My baby slipped the nipple ☐1
    - My baby became frustrated at the breast ☐2
    - My baby fell asleep at the breast ☐3
    - My baby could not open his/her mouth high enough to latch on ☐4
    - My nipples are inverted/flat ☐5
    - My breasts were too engorged ☐6
    - My nipples were sore because of wounds/cracks ☐7
    - Other ☐8
      Please explain**:** ____

26. When was the first time you used the nipple shield for your baby?
    Date: ____________ (approx.)

27. Do you use the nipple shield at discharge?
    - Yes ☐1
    - No ☐2

28. Has your baby used a pacifier during the hospitalization?
    - Yes ☐1
    - No *(proceed to question **33**)* ☐2

29. When was the first time your baby was given the pacifier?
    Date: ___________ (approx.)

30. Did the use of the pacifier change when your infant began to breastfeed more and was less tube-fed?
    - Yes ☐1
    - No *(Proceed to question **32**)* ☐2

31. In what way did the use of the pacifier change?
    - The pacifier was removed completely ☐1
    - The pacifier was predominantly used when I was not present in the ward ☐2
    - The pacifier was predominantly used during nappy changes, blood tests etc. ☐3
    - The pacifier was predominantly used during tube-feedings ☐4
    - Other ☐5
      Please describe**:** ____

32. Does your baby use a pacifier at discharge?
    - Yes ☐1
    - No ☐2

33. Has your baby been bottle-fed during hospitalisation?
    - Yes ☐1
    - No *(proceed to question **36**)* ☐2

34. When was your baby bottle-fed for the first time? Date: ___________ (approx.)

35. Is your baby bottle-fed at discharge?
    - Yes ☐1
    - No ☐2

Questions about skin-to-skin contact

(With skin-to-skin contact we mean that your baby is only dressed in a nappy, maybe a cap and socks, and maybe an open blouse, but in a way that your baby's stomach, chest and legs are in direct contact with your (or another adult's) bare chest.)

36. Have you had your baby skin-to-skin after incubator care?
    - Yes ☐1
    - No *(proceed to question **38**)* ☐2

37. How often has your baby been skin-to-skin after incubator care?
    - A few times ☐1
    - A few times a week ☐2
    - Once a day ☐3
    - Several times a day ☐4

Questions about breast milk pumping, test-weighing and discharge

38. Are you still pumping?

- Yes ☐1
- No ☐2

If no, when did you stop pumping? Date: ________ (approx.)

39. Has your baby been test-weighed during hospitalization?
(With test-weighing we mean weighing the baby just before and just after a breastfeeding session, to calculate the amount of milk the baby has been breastfed)

- Yes, my baby has been test-weighed at most breastfeeding sessions ☐1
- Yes, my baby has been test-weighed a few times ☐2
- No, my baby has not been test-weighed ☐3

40. Have you been at home with your baby before the baby's discharge?

- Yes ☐1
- No *(proceed to question **43**)* ☐2

41. In which way were you at home with your baby before discharge?

- Home visit where we went to the hospital for check-ups ☐1
- Home visits where we got visits from a nurse at home ☐2
- Other: ☐3
  Please describe**:** ____

42. Have you by yourselves given your baby tube-feedings at home?

- Yes ☐1
- No ☐2

43. Are you going for a check-up at the hospital within the first week after discharge?

- Yes ☐1
- No ☐2

44. Do you know who your health visitor is?

- Yes ☐1
- No ☐2

45. Do you have an appointment with your health visitor after discharge?

- Yes ☐1
- No ☐2

46. Do you generally have concerns in connection to discharge?

- No, none *(proceed to question **48**)* ☐1
- Yes, a few concerns ☐2
- Yes, some concerns ☐3
- Yes, a lot of concerns ☐4

47. Please describe any concerns you have:______________________________

____

____

____

____

____

____

The next questions are about whether you have felt you have received sufficient support from your surroundings in your breastfeeding progress. In the questionnaire we ask about practical assistance, encouragement and guidance.
(Please reply with a tick in a box on each line)

48. Have you received sufficient practical help (e.g. bringing things or changing your baby) from the following persons, so you had the time to breastfeed?

a. Your husband/partner	Yes ☐1	No ☐2	Don't know ☐3
b. Your mother/your parents	Yes ☐1	No ☐2	Don't know ☐3
c. Your mother in law/your in-laws	Yes ☐1	No ☐2	Don't know ☐3
d. Others in your family	Yes ☐1	No ☐2	Don't know ☐3
e. Friends	Yes ☐1	No ☐2	Don't know ☐3
f. Nursing staff	Yes ☐1	No ☐2	Don't know ☐3

49. Did you feel you were sufficiently encouraged to breastfeed (e.g. got hugs, a talk or been understood when it was hard) by the following persons?

a. Your husband/partner	Yes ☐1	No ☐2	Don't know ☐3
b. Your mother/your parents	Yes ☐1	No ☐2	Don't know ☐3
c. Your mother in law/your in-laws	Yes ☐1	No ☐2	Don't know ☐3
d. Others in your family	Yes ☐1	No ☐2	Don't know ☐3
e. Friends	Yes ☐1	No ☐2	Don't know ☐3
f. Nursing staff	Yes ☐1	No ☐2	Don't know ☐3

50. Have you received sufficient guidance on breastfeeding (e.g. advice on what to do) by the following persons?

a. Your husband/partner	Yes ☐1	No ☐2	Don't know ☐3
b. Your mother/your parents	Yes ☐1	No ☐2	Don't know ☐3
c. Your mother in law/your in-laws	Yes ☐1	No ☐2	Don't know ☐3
d. Others in your family	Yes ☐1	No ☐2	Don't know ☐3
e. Friends	Yes ☐1	No ☐2	Don't know ☐3
f. Nursing staff	Yes ☐1	No ☐2	Don't know ☐3

Finally, some questions about your baby's hospitalization

51. Has your baby been admitted to several wards?

Yes ☐1
Which ones: ______________________________

No ☐2

52. Has your baby been on a ventilator?

Yes ☐1
No ☐2

If yes: for how many days? approx. ______ days

53. Has your baby been treated with nasal CPAP?

Yes ☐1
No ☐2

If yes: for how many days? approx. ______ days

54. Has your baby had an ultrasound examination of the head?

Yes ☐1
No *(proceed to question **56**)* ☐2

55. If yes: what did the examination show:

It was normal ☐1
It was not normal ☐2

56. Have there, during the hospitalization, been particular problems with your baby's health (e.g. surgery, congenital defects or other serious illness)?

Yes ☐1
No ☐2

If yes, please describe

____

____

____

____

____

If you like, you can note what has meant the most to you with regard to breastfeeding – positively and negatively. You are welcome to come up with some examples.
You are also welcome to enter any comments or suggestions for changes/improvements that could have made your breastfeeding process during the hospitalization better.
Please write on the following lines (and, if you wish, on the following pages).

Thank you for taking your time to answer the questionnaire.

If, at your baby's discharge, you are expressing breast milk, breastfeeding fully or partially, you will be called by telephone 1, 4, 6 and 12 months after your baby's estimated date of delivery, in order to know if you are still breastfeeding or expressing breast milk.
If, at discharge, you are not breastfeeding or expressing breast milk you will not be contacted again.

Please return the questionnaire to the staff.

The survey is done in cooperation with
Knowledge Centre for Breastfeeding Infants with Special Needs
Copenhagen University Hospital, Rigshospitalet
Department of Neonatology
Copenhagen
Denmark
