# Supplementary material for: Factors Associated with Exclusive Breastfeeding of Preterm Infants. Results from a Prospective National Cohort Study
Source: PLoS One. 2014 Feb 19;9(2):e89077. doi: 10.1371/journal.pone.0089077 (PMC3929624; doi:10.1371/journal.pone.0089077)
Supplement: Questionnaire S3 — Breastfeeding survey. Questionnaire 3 used for telephone interviews with the baby’s mother. (PDF) [file pone.0089077.s003.pdf]

Ward number _____ Code number ________

Breastfeeding study of preterm infants in neonatal wards in Denmark 2009 – 2011

Questionnaire 3

Telephone interview with the baby's mother

Follow-up

Telephone interview by: _____________________ Date: ___________

We are phoning you/writing to you because you are participating in "The breastfeeding survey of preterm infants", and we hope you have time to answer the following questions.

---

1. Your baby's corrected age (age after term):
   - 1 month ☐1
   - 4 months ☐2
   - 6 months ☐3
   - 12 months ☐4

2. Are you still breastfeeding?
   - Yes, my baby is fully breastfed* *(Proceed to question **5**)* ☐1
   - Yes I am breastfeeding, but my baby is also getting something else ☐2
     *(Go to question **3**)*
   - No, I am not breastfeeding any longer ☐3
     *(Proceed to question **4**)*

3. When did your baby start being fed in a way other than breastfeeding?
   Approx. date ___________

   a. What is your baby getting besides breastfeeding?
   (You may tick more than one box)
   - Pumped breast milk ☐1
   - Infant formula ☐2
   - Solid foods ☐3
   - Gruel ☐4

   b. What was the reason(s) that you stopped full breastfeeding?
   ____
   ____
   ____
   ____
   ____

---

* With full breastfeeding we mean that the baby is only fed at and from the breast. In Denmark that means, that besides breastfeeding, the baby can be given water and/or a maximum of one formula feed a week.

4. When did you completely stop breastfeeding?

Date____________(approx.)

a. What is your baby getting instead of breastfeeding?
(You may tick more than one box)

- Pumped breast milk ☐1
- Infant formula ☐2
- Solid foods ☐3
- Gruel ☐4

b. What was the reason(s) that you completely stopped breastfeeding?

____
____
____
____
____

The following questions are about the use of a nipple shield.

5. Did you use a nipple shield for breastfeeding your preterm infant?
   - Yes ☐1
   - No *(Proceed to question **9**)* ☐2

6. When did you begin using the nipple shield?
   - In hospital ☐1
   - After discharge ☐2

7. Are you still using the nipple shield?
   - Yes *(Proceed to question **9**)* ☐1
   - No ☐2

8. If no, when did you stop using the nipple shield? Date: ________ (approx.)

Some questions about breast milk pumping.

9. Did you continue to pump breast milk after discharge?
   - Yes ☐1
   - No *(Proceed to question **12**)* ☐2

10. Are you still pumping?
   - Yes *(Proceed to question **12**)* ☐1
   - No ☐2

11. If no, when did you stop breast milk pumping? Approx. date: ________

The last questions are about breastfeeding problems

12. Have you experienced breastfeeding problems (since last questionnaire/contact)?
   - Yes ☐1
   - No ☐2

Which problems: ____
____
____
____
____
____
____

13. How would you describe your breastfeeding after discharge?
   - Mainly functioning ☐1
   - Mainly problematic ☐2

Thank you for taking your time to answer the questionnaire.
If you are not breastfeeding or pumping any more, we will not contact you again.
